# The WHO 2016 verbal autopsy instrument: An international standard suitable for automated analysis by InterVA, InSilicoVA, and Tariff 2.0

**DOI:** 10.1371/journal.pmed.1002486

**Published:** 2018-01-10

**Authors:** Erin K. Nichols, Peter Byass, Daniel Chandramohan, Samuel J. Clark, Abraham D. Flaxman, Robert Jakob, Jordana Leitao, Nicolas Maire, Chalapati Rao, Ian Riley, Philip W. Setel, on behalf of the WHO Verbal Autopsy Working Group

**Affiliations:** 1 National Center for Health Statistics, Centers for Disease Control and Prevention, United States Public Health Service, Hyattsville, Maryland, United States of America; 2 WHO Collaborating Centre for Verbal Autopsy, Umeå Centre for Global Health Research, Division of Epidemiology and Global Health, Department of Public Health and Clinical Medicine, Umeå University, Umeå, Sweden; 3 MRC-Wits Rural Public Health and Health Transitions Research Unit (Agincourt), School of Public Health, Faculty of Health Sciences, University of the Witwatersrand, Johannesburg, South Africa; 4 London School of Hygiene & Tropical Medicine, London, United Kingdom; 5 Department of Sociology, The Ohio State University, Columbus, Ohio, United States of America; 6 ALPHA Network, London School of Hygiene and Tropical Medicine, London, United Kingdom; 7 INDEPTH Network, Accra, Ghana; 8 Institute for Health Metrics and Evaluation, Department of Global Health, University of Washington, Seattle, Seattle, Washington, United States of America; 9 World Health Organization (WHO), Geneva, Switzerland; 10 Department of Epidemiology and Public Health, Swiss Tropical and Public Health Institute, Basel, Switzerland; 11 University of Basel, Basel, Switzerland; 12 Department of Global Health, Research School of Population Health, Australian National University, Canberra, Australia; 13 Melbourne School of Population and Global Health, University of Melbourne, Melbourne, Australia; 14 Vital Strategies, New York, New York, United States of America

## Abstract

**Background:**

Verbal autopsy (VA) is a practical method for determining probable causes of death at the population level in places where systems for medical certification of cause of death are weak. VA methods suitable for use in routine settings, such as civil registration and vital statistics (CRVS) systems, have developed rapidly in the last decade. These developments have been part of a growing global momentum to strengthen CRVS systems in low-income countries. With this momentum have come pressure for continued research and development of VA methods and the need for a single standard VA instrument on which multiple automated diagnostic methods can be developed.

**Methods and findings:**

In 2016, partners harmonized a WHO VA standard instrument that fully incorporates the indicators necessary to run currently available automated diagnostic algorithms. The WHO 2016 VA instrument, together with validated approaches to analyzing VA data, offers countries solutions to improving information about patterns of cause-specific mortality. This VA instrument offers the opportunity to harmonize the automated diagnostic algorithms in the future.

**Conclusions:**

Despite all improvements in design and technology, VA is only recommended where medical certification of cause of death is not possible. The method can nevertheless provide sufficient information to guide public health priorities in communities in which physician certification of deaths is largely unavailable.

The WHO 2016 VA instrument, together with validated approaches to analyzing VA data, offers countries solutions to improving information about patterns of cause-specific mortality.

## Abstract 2

Erin Nichols and colleagues describe the development of the WHO verbal autopsy standard instrument

## Background

**Fig 1:**
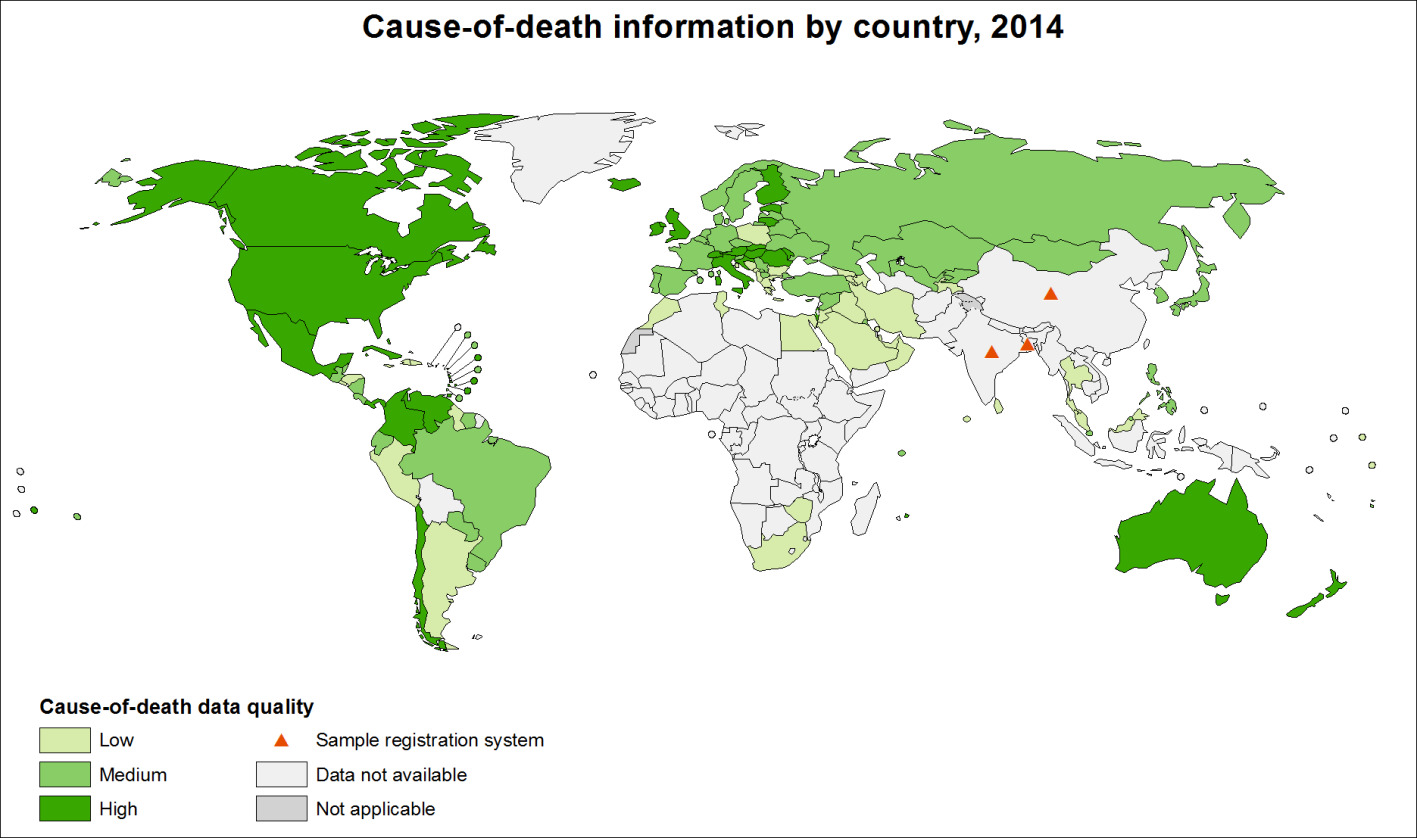
Cause-of-death information by country, 2014 [3]. The boundaries and names shown and the designations used on this map do not imply the expression of any opinion whatsoever on the part of the World Health Organization concerning the legal status of any country, territory, city or area or of its authorities, or concerning the delimitation of its frontiers or boundaries. Dotted and dashed lines on the map represent approximate border lines for which there may not yet be full agreement. Data Source: World Health Organization Map Production: Public Health Information and Geographic Information Systems (GIS) World Health Organization.

In low-income countries, many deaths are unregistered, unrecorded, and unnoticed by the health system. Nearly half of all countries fail to meet United Nations standards for death registration (90% coverage) [1], while high-quality cause-of-death data are lacking for 65% of the world’s population (see Fig 1) [2,3]. Inadequate data on cause-specific mortality patterns impede the development of sound health policy, planning, monitoring, and evaluation [4].

Where medical certification of cause of death is not possible and civil registration and vital statistics (CRVS) systems are weak, verbal autopsy (VA) has been introduced as a practical method for determining probable cause of death [5]. VA involves a structured interview with the next of kin or a caregiver of the deceased after a mourning period about signs and symptoms the deceased experienced before death.

There are multiple VA instruments, diagnostic methods, and analysis procedures [6]. Methods suitable in routine settings have developed in the last decade, including automated methods for assigning cause of death. Developments have been paralleled by a growing global momentum to strengthen CRVS systems in low-income countries, and global partners have agreed on the need for a single standard VA instrument on which multiple diagnostic methods can be developed [7], working towards a global standard for reporting VA results. In 2016, partners harmonized a WHO VA standard instrument that incorporates indicators necessary to run available automated diagnostic algorithms alongside conventional physician review for assignment of causes of death. This paper describes key developments of the WHO 2016 VA instrument as a harmonized international standard.

### Introduction of an international standard VA instrument

In 2007, WHO introduced the first international technical standards and guidelines for VA, following recommendations from a 2005 systematic review of the most widely used instruments and procedures [8]. The VA standard instrument included a separate questionnaire for 3 age groups—under 4 weeks, 4 weeks to 14 years, and 15 years and older—in addition to a cause-of-death list for VA with corresponding codes from the 10th revision of the International Statistical Classification of Diseases and Related Health Problems (ICD-10) [9]. The cause-of-death list indicates the degree of specificity of the VA instrument for different categories of causes (e.g., cancers, external causes, maternal causes, and perinatal causes, including stillbirths). In 2012, WHO published a simplified VA instrument [10]. The aim of the simplification process was to develop an instrument for routine use, including in the context of a national CRVS system. Compared to the 2007 instrument, questions were modified to facilitate a dichotomous yes/no response (or some multiple select values) and to capture continuous variables with their value, and the overall number of conditions and questions was reduced. This process marked a significant shift from the previous uses of VA, which were generally limited to small-scale research and surveillance settings.

A key modification critical for routine VA use is automating the analysis process to reduce clinician burden in reviewing questionnaires. An iterative process of modifications was initiated in 2014 to (1) to address recommendations from field experience and cognitive testing of the 2012 instrument that was conducted in western Kenya to review the measurement validity of the questionnaires [11] and to (2) add or edit questions to facilitate the use of publicly available automated analytical software for assigning cause of death [12]. In 2016, questions were again added or edited to reach full compatibility with the available automated analysis methods. For the WHO VA questionnaire, while a valid measurement tool is a priority, input from the performance of compatible algorithms is informative in the questionnaire revision and improvement process. Thus, the questionnaire development process now involves an iterative review process, balancing measurement performance with algorithm compatibility. This compatibility is the key feature of the WHO 2016 VA instrument described below.

## Harmonization of the WHO 2016 VA instrument

### Consensus process

The authors of this paper comprised a panel convened in 2016 to harmonize through consensus the components required for compatibility with 3 currently available automated diagnostic methods—namely, InterVA [13], InSilicoVA [14], and Tariff 2.0 [15]. Participants were selected based on their involvement in the development of the WHO 2012/2014 or Population Health Metrics Research (PHMRC) instruments or analysis methods. All invited participants participated. Since InterVA-4 and InSilicoVA were designed to be fully compatible with the WHO 2012 standard, the consensus meeting focused on Tariff 2.0 compatibility. Consensus was defined as the WHO VA questionnaires including all content collected by the PHMRC questionnaires, as well as retaining the WHO 2012 VA content, and ensuring that remaining discrepancies in wording did not reflect differences in the meaning of the related questions. Prior to the meeting, participants listed any discrepancies preventing compatibility. Each discrepancy was discussed one by one; a satisfactory solution was reached for remaining discrepancies, either by adding a necessary indicator or modifying the phrasing or order of existing indicators. The consensus meeting addressed all discrepancies and issues associated with the key objective of harmonizing the WHO VA instrument for use with the 3 analysis methods. While there were no remaining discrepancies preventing compatibility after the meeting, a list of issues was marked for further review and consideration at future meetings, once additional information on instrument performance is available.

### WHO 2016 VA instrument

The WHO 2016 VA and PHMRC questionnaires have similar structures [16]. There are 3 questionnaires specific to different age groups: for perinatal and neonatal deaths, children aged under 4 weeks; for postneonatal and child deaths, children aged 4 weeks to 11 years; and for adult deaths, adults aged 12 years or older. Some questionnaire sections are common to all 3 age groups, while others are specific to certain age groups. The general structure of all 3 questionnaires includes information about the date and location of the interview; the field site and household; the primary respondent; sociodemographics of the deceased; a history of injuries/accidents; a symptom duration checklist; health services used by the deceased during illness in the period before death, including whether a health worker informed the respondent of the cause of death; medical death certificate (if available); a summary of any medical evidence available at the household; and an open narrative history of events leading to death, with a checklist of key conditions indicated as present in the narrative, and the cause-of-death according to the respondent. The open narrative provides critical information for physician review of VA, while the checklist of key conditions is used to increase the accuracy of Tariff 2.0 analysis [17]. In addition to general questions, each questionnaire contains sections and questions specific to the circumstances of the death, including a series of questions involving symptoms and their duration. These questions are different for each questionnaire and are the essence of the VA tool [16].

A first release candidate of the WHO 2016 VA instrument was published online on 30 January 2017. The first release candidate has been carefully reviewed and field tested by teams in South Africa, Kenya, Mozambique, and Morocco. Reported issues have been reviewed and addressed by the WHO VA Working Group, and a second release candidate is now available [16].

The WHO 2016 VA instrument is available for download in the form of an Excel template suitable for electronic data collection using the Open Data Kit (ODK) platform (https://opendatakit.org/) [17] or compatible products. Features including automatic skip patterns, constraints, and range and logic checks have been added to optimize use of the electronic data collection platform. The WHO 2016 VA instrument contains a superset of the variables required by the publicly available analytical software for assigning cause of death. The WHO 2016 VA instrument comes with a guide that explains the background, structure, intended use, and principles for implementing VA. A package of guidance materials is being developed to support implementation of the WHO 2016 VA instrument. These materials include an interviewer manual, including specific question-by-question instructions; a VA supervisor’s manual; a technical administrator and user manual with guidance on data use and interpretation; a guide to setting up a VA system; and release notes highlighting detailed differences between the 2012, 2014, and 2016 versions. These materials are being made available for download on the WHO website (see also S1 Table, “Summary comparison of verbal autopsy (VA) questionnaires”) [16].

## Analysis processes compatible with the WHO 2016 VA instrument

Three automated diagnostic algorithms that are freely available, have been evaluated for acceptable performance, are compatible for use with the WHO 2016 VA instrument, and can be used in routine CRVS systems are briefly described below. These automated methods and physician review, the traditional method for analyzing VA data, are also described.

### InterVA and InSilicoVA automated diagnostic algorithms

The InterVA algorithm [13] was developed and revised over a number of years starting in 2003. Based on Bayes’ rule for conditional probabilities, for a single death InterVA produces values for the propensity of each cause given the indicators reported as present in a VA interview and a set of evidence-based and physician-derived conditional probabilities describing the typical likelihood of each indicator for deaths of each cause. For a set of deaths, InterVA sums across the largest propensities for each cause to yield the population-level fraction of deaths resulting from each cause. For each death, InterVA reports single value point estimates for the propensity of the 3 causes with the largest propensities, if they fall above a set threshold; otherwise, the cause is ruled “indeterminate.” Full details, source code, and compiled executables that implement InterVA-4 (version 4.04) are available at http://www.interva.net. When WHO established the WHO 2012 VA standard, InterVA was updated to version 4, which uses all 254 WHO 2012 VA indicators and codes deaths to all 62 ICD-based cause categories defined in WHO 2012 [13]. InterVA-4 has been used on a large scale, including assigning causes to deaths in the INDEPTH Network’s public-domain VA dataset [18], and was also implemented and evaluated in an experimental mobile version, which included real-time attribution of cause of death [19]. To accommodate the WHO 2016 standard, InterVA-5 has been developed and is being tested. InterVA-5 utilizes all WHO 2016 indicators and codes to all 63 WHO 2016 causes; in addition, it is able to accept subsets of VA indicators produced by the WHO 2012 standard or PHMRC instrument.

InSilicoVA [14] is a statistical algorithm that, for a set of deaths, identifies the most likely joint probability distribution of cause-specific mortality fractions and probabilities of each cause for each individual death. This is done using a Bayesian hierarchical model fit using a Gibbs sampling algorithm that uses information on both the presence and absence of VA indicators and the conditional probability of each VA indicator for deaths of each cause. Those conditional probabilities are interchangeable and can be borrowed from InterVA-4, calculated from the PHMRC gold-standard dataset (see below), or come from another source. InSilicoVA reports probability distributions and summaries of those distributions for each cause-specific mortality fraction and the probability of each cause for each death. This is a first step in accounting for the inherent uncertainty in assigning causes to deaths using VA. The current version of InSilicoVA supports the WHO 2012 standard VA indicators and cause list, identical to InterVA-4. Free, open-source software (including source code) implementing InSilicoVA is available for the R statistical programming language—download at https://CRAN.R-project.org/package=InSilicoVA. The performance of InSilicoVA has been compared to InterVA-4, Tariff 1.0, and other less used VA coding algorithms using both the InterVA-4 conditional probabilities and similar probabilities calculated from the PHMRC gold-standard deaths. All algorithms were used to assign causes to the PHMRC gold-standard deaths, and the cause assignments were compared to the medically certified causes in that dataset. The performance of each algorithm was quantified and compared to the other algorithms [14]. As soon as the WHO 2016-compatible InterVA-5 is available, InSilicoVA will be updated to be able to use the InterVA-5 conditional probabilities and WHO 2016 cause list so that it will be fully compatible with the WHO 2016 standard.

### SmartVA and Tariff 2.0

In 2005, concurrent to the development and revision of the WHO standard VA instrument, the PHMRC initiated a VA validation study. In this study, 12,542 VA interviews were conducted for which a gold-standard underlying cause of death was known (based on clinically reliable diagnostic criteria) [20]. As part of this study, the PHMRC developed a VA questionnaire (PHMRC Full Questionnaire) based on the WHO 2004 VA Technical Consultation on Verbal Autopsy Tools [8], with modifications suggested by previous experience, other validated or widely used instruments, and expert judgement [20]. The validation data collected with the PHMRC Full Questionnaire were used to compare all known methods of automated analysis of VA with each other and with novel methods [6]. This exercise led to the development of the Tariff method [21], an approach that balances data-driven machine-learning methods with a level of interpretability necessary for methodological acceptance (this balance is coming to be known as “explainable artificial intelligence”). The Tariff method produces a set of tariff scores for each disease–symptom pair, which can be interpreted as a robust analogue to a z-score and indicate the relatedness of each symptom to each cause.

Application of VA in routine CRVS requires as short a questionnaire as possible. In order to derive a minimal questionnaire from the lengthier instrument, the PHMRC undertook a data-driven item reduction exercise to identify questions that contribute little to identifying causes so that they could be dropped from the questionnaire [22]. The tariff scores were used to rank order the questions of the PHMRC Full Questionnaire from most informative to least, and an in silico experiment with the PHMRC gold-standard validation data was conducted to find a suitable cutoff that separated informative from uninformative questions—i.e., those that could be removed without substantially affecting the accuracy of the Tariff method—and the uninformative questions were dropped. This exercise resulted in the PHMRC Shortened Questionnaire [22]. Subsequent testing in additional field sites identified additional improvements to the Tariff method, which have been implemented in the Tariff 2.0 version [15]. Dubbed “SmartVA,” the PHMRC Shortened Questionnaire and Tariff 2.0 were packaged for implementation at scale using handheld devices (e.g., using tablets or smartphones) and programmed with interview software based on the ODK platform (https://opendatakit.org/) [17]. SmartVA software is available at http://www.healthdata.org/verbal-autopsy/tools.

### Physician review-based assignment of causes of death

Physician review of completed questionnaires has been the convention for assigning causes of death from VA. Since 2005, a standardized approach to apply physician review in VA has been developed and applied in VA programs in several countries [23–29]. In this approach, physicians trained in standard international practices for cause-of-death certification carefully review completed VA questionnaires, collate information from various sections of the form, and apply clinical judgement in conformation with prescribed diagnostic algorithms for specific conditions, to assign probable causal sequences of diseases or conditions leading to death, and any associated or contributory factors [9]. Subsequently, specific rules prescribed by ICD-10 are applied to select and code the underlying cause(s) of death [9]. From a theoretical perspective, physician review represents a direct, clinically plausible, and readily understood and directly verifiable method for ascertaining causes of death from VA. However, standardization of physician review protocols, rigorous training programs, data quality control mechanisms, and regular assessment of reliability and validity of physician cause attribution from VA are needed to assure data quality. All of these elements have resource implications for implementation in a routine program for assessment of causes of death in a population. Physician review is expected to remain a core element of VA development as a quality control mechanism.

## Discussion

The WHO 2016 VA instrument provides a base for continued research and development. Refinement is needed to shift from emerging to best-practice VA processes, including a cause-of-death coding mechanism, for routine use. This refinement process will continue to balance efficiency, effectiveness, and affordability. Efforts to streamline VA processes can be supported by advances in “big data science” and in leveraging new machine-learning/data-driven approaches. The WHO 2016 VA instrument will provide a common platform to collect VA data in a standard way to synthesize the evidence and practical experience in order to refine the questionnaires and cause-of-death assignment processes and to support validation and performance review at the country level.

Questionnaire design is an example of the need to bring together data-driven methods and real-world understanding—particularly for an international standard instrument—because the questions asked will influence the knowledge base going forward. One shortcoming to the review process at this stage of VA development is the requisite inclusion of all indicators in the questionnaire to create compatibility across multiple analytic methods. Performance testing of the resulting questionnaire will facilitate a comprehensive item reduction process, which will be a key focus of the next instrument revision. Until then, the current WHO VA questionnaire will temporarily remain longer than is ideal for implementation on a routine basis.

Several additional areas were flagged for further investigation, including further review of wording and ordering of select questions, implementation guidelines, how to harmonize causes of death reached by different algorithms and information obtained from multiple sources, and implications for legal and administrative uses of VA data within a CRVS system. Critically, further guidance is needed on how to interpret and use potentially divergent output from different analysis processes and in what contexts the various processes should be used. These issues will be further investigated in future work of the WHO VA Working Group.

We can expect further developments in the WHO VA standards in 2 to 3 years. Future revisions will be informed by implementation research, validation processes, and international comparative data analyses. Medical certification of cause of death remains the gold-standard method for determining cause of death. However, where medical certification is not possible, the WHO 2016 VA instrument, together with validated approaches to analyzing VA data, offers countries solutions to improving information about patterns of cause-specific mortality.

## Supporting information

S1 Table Summary comparison of verbal autopsy (VA) questionnaires. (XLSX)

## Abbreviations

CRVS: civil registration and vital statistics
ICD-10: 10th revision of the International Statistical Classification of Diseases and Related Health Problems
ODK: Open Data Kit
PHMRC: Population Health Metrics Research
VA: verbal autopsy
